# The Story of the Insane from Year to Year

**Published:** 1895-09-14

**Authors:** 


### THE STORY OF THE INSANE FROM YEAR TO YEAR.

### CHESTER COUNTY ASYLUM AT UPTON.

This asylum began the year with 600 patients, and ended it with 617, the average daily number resident being 614. There were 165 admissions, 66 recoveries, and 65 deaths. Calculated on the admissions, the percentage of recoveries is 41·5, and calculated on the average number resident the percentage of deaths is 10·5, both of these being a trifle above the average in kindred institutions. Of the deaths 17 were owing to cerebral diseases either alone or in combination with other diseases, 15 were caused by consumption, and 1 by enteric fever. Turning to Table X., we find that intemperance in drink caused the insanity in 23 of the admissions, domestic trouble in 3, religion in 3, and hereditary influence in 40. Like so many of his brother superintendents, Dr. Davidson bewails the class of patients sent to him for treatment, the majority presenting little hope of recovery, and on the first day of the new year there were only 19 of the 600 of whom any reasonable hope of recovery could be entertained. We notice that a detached hospital for the treatment of infectious disease has been built at a cost of £965. This seems extremely cheap, and we doubt whether a properly planned and equipped hospital can be built for the money. There was an outbreak of enteric fever, and Dr. Davidson says that "After diligent searching and careful investigation to discover the cause of the malady, I am unable to point to any marked defect in the sanitation of the asylum." The committee say they "have much pleasure in again recording their great satisfaction with the economical and efficient manner in which Dr. Davidson and his staff have managed the asylum." The average weekly cost per head is only 6s. 6½d., and making every allowance for the cheapness of provisions, we cannot help thinking that it is too low.

### ROYAL ASYLUM, EDINBURGH.

On January 1st, 1894, there were 843 patients on the books of this asylum, and by the end of the year this number had risen to 882. There were 454 admissions, 171 discharged recovered, 133 discharged relieved, and 85 deaths. The recoveries stand at 37·7 per cent. of the admissions, and the deaths at 6·6 per cent. of the average number resident. Cerebral and spinal diseases alone or in combination with other diseases account for 68 of the deaths, and consumption for 4. Intemperance in drink accounts for 83 of the admissions; mental anxiety and domestic troubles for 24, and hereditary influence for 118. Whether insanity is increasing at present in a higher proportion than the increase of the population accounts for is a disputed point. Dr. Clouston thinks not, and it is consoling to find him saying that his "own investigations seem to prove definitely that on this point there is little cause for alarm." The great event of the year was the opening of the new buildings known as Craig House, and a short description of it is given in Dr. Clouston's report. He says that "all that taste, colour, and art could do has been called in for the benefit and cure of what was once regarded as a disease to be shunned and loathed." A few years since a vast deal of nonsense was talked about hospitals for the treatment of the presumably curable insane, and we were treated to a variety of predictions concerning the advantages which would accrue from the institution of these adjuncts to our asylums. This journal never shared in these optimistic views. It was known that they had been tried on the Continent and elsewhere, and that the marvellous results had not been forthcoming. Dr. Clouston tells us there is now a reaction on the Continent. It was found that sharp lines between the curable and incurable did not work well in practice, and were not for the happiness and best interests of either class. There is, therefore, a reversion setting in to the older mixed institutions. It is extremely pleasing to us to note that the charitable side of this asylum is kept steadily in view, and Dr. Clouston regrets he is not oftener able to admit patients whose friends can afford low rates of payment only. The managers have been able to reduce the charges for this class to £30 per annum, and intermediate patients are admitted at £42 per annum.

